# Supplementary material for: Chromothripsis during telomere crisis is independent of NHEJ, and consistent with a replicative origin
Source: Genome Res. 2019 May;29(5):737–49. doi: 10.1101/gr.240705.118 (PMC6499312; doi:10.1101/gr.240705.118)
Supplement: Supplemental Material [file supp_gr.240705.118_Supplemental_file_1.zip › contigs/annotated_contigs/DB116/contig.2.DB116_length_702_mean_cov_17.3988603989.docx]

**DB116_length_702_mean_cov_17.3988603989**

```
AGACACAGATACTCATAATGTTCAGAATCAGAAAAGGCCTCTACAATATAGAATATAGTTCCAGGAATAAGTTCATTTCTCCATGGTTA
 >chr11:38096388-38096753 + E=8e-208 p=2e-02
TCTCTTTCTGAAATCTTTGTCTGTCTAACCTATTACTTCAGCAGCTTTCTGATGATTTTATACAGATTTTCACTATTTGTTTCTATATA

GCTTTTCGAGATTAAGGAGAGAGTATTTGTGTATTTTAAGTTTCTCCAGCAAAAAGCCAAGCTTTGTATTTGTCATTGACTTTTGATCA

ATACTTTCAGCATATTTCCAACAAAGAGTCTATATGCATAGCCCACCAGGATTTCATGAAAGTACCTATTTTCCATCAATGTAATCAGC

ATTG|GATAT|GATGAATATTTCATGGGTATTGCATGAATCGTGAACAAAAGCAATGTCAATAGAGACAAAAAATATTTTTGTTTCTAT
     >chr11:38097301-38097643 + E=6e-191
TTCAGTTTCTGGTTTAGAGAACTGAATTTATGCAAGGAAAATTTTCTGCTCCTATATAATGGAATCAATTGAAATCCATGTATGTAATT

GCAAAGACGTATAAGATCAAAAGATGTCATAAATGATAGATTTTCTTACATTAGTTTAATGCACCATGCTCTCATGAAATAAAGTTAAC

CAAGATAAAGATTTCACCAATTCTTGTCTTTTAAAAAATATTTCATAGTCATTTATAATCTTGGACCTGGAATCTGGAATT
```
